# A pilot study on the usefulness of peripheral blood flow cytometry for the diagnosis of lower risk myelodysplastic syndromes: the “MDS thermometer”

**DOI:** 10.1186/s12878-018-0101-8

**Published:** 2018-03-13

**Authors:** Ana Aires, Maria dos Anjos Teixeira, Catarina Lau, Cláudia Moreira, Ana Spínola, Alexandra Mota, Inês Freitas, Jorge Coutinho, Margarida Lima

**Affiliations:** 1 Department of Hematology, Hospital de Santo António (HSA), Centro Hospitalar do Porto (CHP), Porto, Portugal; 2 Department of Pathology, Hospital de Santo António (HSA), Centro Hospitalar do Porto (CHP), Porto, Portugal; 3 Instituto de Ciências Biomédicas Abel Salazar, Universidade do Porto (ICBAS/UP), Porto, Portugal; 4 Unidade Multidisciplinar de Investigação Biomédica, Instituto de Ciências Biomédicas Abel Salazar, Universidade do Porto (UMIB/ICBAS/UP), Porto, Portugal; 5 Laboratório de Citometria, Serviço de Hematologia, Hospital de Santo António, Centro Hospitalar do Porto, instalações do Ex-CICAP, Rua D. Manuel II, s/n, 4099-001 Porto, Portugal

**Keywords:** Myelodysplastic syndromes, Lower risk MDS, Peripheral blood, Flow cytometry

## Abstract

**Background:**

Immunophenotypic analysis of the bone marrow (BM) cells has proven to be helpful in the diagnosis of Myelodysplastic Syndromes (MDS). However, the usefulness of flow cytometry (FCM) for the detection of myelodysplasia in the peripheral blood (PB) still needs to be investigated. The aim of this pilot study was to evaluate the value of FCM-based PB neutrophil and monocyte immunophenotyping for the diagnosis of lower risk MDS (LR-MDS).

**Methods:**

We evaluated by 8-color FCM the expression of multiple cell surface molecules (CD10, CD11b, CD11c, CD13, CD14, CD15, CD16, CD34, CD45, CD56, CD64 and HLA-DR) in PB neutrophils and monocytes from a series of 14 adult LR-MDS patients versus 14 normal individuals.

**Results:**

Peripheral blood neutrophils from patients with LR-MDS frequently had low forward scatter (FSC) and side scatter (SSC) values and low levels of CD11b, CD11c, CD10, CD16, CD13 and CD45 expression, in that order, as compared to normal neutrophils. In addition, patients with LR-MDS commonly display a higher fraction of CD14^+^CD56^+^ and a lower fraction of CD14^+^CD16^+^ monocytes in the PB. Based on these results, we proposed an immunophenotyping score based on which PB samples from patients with LR-MDS could be distinguished from normal PB samples with a sensitivity 93% and a specificity of 100%. In addition, we used this score to construct the MDS Thermometer, a screening tool for detection and monitoring of MDS in the PB in clinical practice.

**Conclusions:**

Peripheral blood neutrophil and monocyte immunophenotyping provide useful information for the diagnosis of LR-MDS, as a complement to cytomorphology. If validated by subsequent studies in larger series of MDS patients and extended to non-MDS patients with cytopenias, our findings may improve the diagnostic assessment and avoid invasive procedures in selected groups of MDS patients.

**Electronic supplementary material:**

The online version of this article (10.1186/s12878-018-0101-8) contains supplementary material, which is available to authorized users.

## Background

Myelodysplastic Syndromes (MDS) are a group of myeloid neoplasms characterized by inefficient hematopoiesis, peripheral blood (PB) cytopenias and high risk of leukemic progression [1–3]. According to World Health Organization (WHO) classification, lastly updated in 2016, the diagnosis is essentially based on morphological and cytogenetic abnormalities, such as the presence of cytopenias, blasts in the PB and/or bone marrow (BM), and dysplasia in one or more hematopoietic cell lineages [4, 5]. Although PB cytomorphological findings provide information to suspect of MDS, only BM studies are presently accepted to confirm the diagnosis [4, 5].

Five major MDS subtypes are currently recognized (WHO, 2016): MDS with single lineage dysplasia (MDS-SLD), MDS with multilineage dysplasia (MDS-MLD), MDS with ring sideroblasts (MDS-RS), MDS with isolated deletion of chromosome 5q [MDS-del(5q)], MDS with excess of blasts (MDS-EB), and MDS, unclassifiable (MDS-U) [5]. The International Prognostic Scoring System (IPSS), has been used to estimate risk for progression to acute myeloid leukemia (AML) or death from cytopenia-related complications. [6]. Patients categorized as low or intermediate-1 risk using the IPSS are usually referred to as “lower-risk” MDS (LR-MDS), whereas those classified as intermediate-2 or high risk are usually termed “higher-risk” MDS (LR-MDS). In its revised version, the IPSS incorporated new BM blast classes and cytogenetic abnormalities, and included both number and severity of cytopenias, thereby defining five (very low, low, intermediate, high and very high) risk categories, from which the first three correspond to LR-MDS [7]. In general, these include MDS-SLD, MDS-MLD, MDS-RS and MDS-del(5q).

Most of the LR-MDS patients, who account for around 60% of newly diagnosed MDS cases, remain simply on supportive care, being dependent on red blood cell (RBC) and/or platelet transfusions, and/or receiving hematopoietic growth factors; about 1/3 of the patients only require monitoring (“wait and see”) [8]. HR-MDS patients may benefit from intensive treatments, although most of them are not eligible due to increased age and/or comorbidities, thereby being selected for low-intensity treatment regimens [8].

Flow cytometry (FCM) is a highly sensitive technique for evaluation of the hematopoietic cells. It has been used with increasing frequency to study the BM from patients with MDS, being considered a promising tool to improve MDS diagnosis, especially in cases of minimal dysplasia, absence of cytogenetic abnormalities, and BM hypocellularity or fibrosis [9–16]. Its value in the diagnosis of Chronic Myelomonocytic Leukemia (CMML) and other Myelodysplastic/myeloproliferative neoplasms (MDS/MPN) has also been documented [17–20].

Flow cytometry has an increasing importance in MDS diagnosis and subtyping, and in predicting the clinical outcome [15, 21]. However, a systematic histological and immuno-histochemical examination of the BM is still required for the final diagnosis and classification of MDS [22].

Several immunophenotypic abnormalities have been reported by FCM in the BM from MDS patients. Some examples are increased numbers of CD34^+^ precursors, abnormal expression of cell surface molecules on myeloblasts, maturing granulocytic and monocytic cells, or erythroid precursors, and lineage infidelity [13]. For instance, phenotypic abnormalities of CD34^+^ cells and their compartments have been reported in MDS, with LR-MDS patients typically having an expansion of myeloid CD34^+^ cells at the expense of lymphoid B-cell precursors, while expansion of immature CD34^+^ cells occurs in HR-MDS [11, 23]. Aberrant antigen expression (e.g., CD5, CD7 and CD56), and over or under expression of other cell surface markers (e.g. CD13, CD34, CD45, CD117 and HLA-DR) on CD34^+^ myeloblasts have also been reported [12, 24].

Asynchronous shift to the left in maturing granulocytes is also frequent in the BM from patients with MDS, with neutrophil-precursors and maturing neutrophils having decreased size and granularity, and, consequently, a lower light scatter. Abnormal/Asynchronous expression of CD11b, CD13, CD15 and CD16 molecules has also been described, reflecting an anomalous neutrophil maturation [9, 10, 12]. Likewise, BM monocytes from MDS patients frequently have abnormal maturation patterns, as evaluated by the expression of CD14, CD34, CD36, CD64, and HLA-DR. Erythroid dysplasia has also been documented by FCM, by studying a set of molecules that are expressed differently throughout the maturation of RBC in the BM, such as CD35, CD36, CD44, CD45, CD71, CD105, CD117 and CD235a [9, 12, 25, 26].

Even though the collection of a PB sample is much simpler and much less invasive than a BM aspirate and/or biopsy, nearly all FCM studies in MDS patients have been performed in BM samples; the immunophenotypic alterations in PB cells have been much less explored [27–31].

Taking in consideration the accessibility of PB samples, it would be useful to establish FCM criteria for the diagnosis of myelodysplasia in the PB, especially in patients with LR-MDS. Therefore, the purpose of this study was to search the presence of abnormal and/or aberrant antigen expression in circulating neutrophils and monocytes from these patients. Based on the results obtained, a straightforward FCM-based scoring system is proposed, which allows to distinguish PB samples of patients with LR-MDS from normal PB samples with a high sensitivity and specificity. Using this scoring schema we, conceived the MDS Thermometer, a simple screening tool for detection and monitoring of MDS in the PB in clinical practice.

## Methods

### Study population and design

This study included 14 patients with LR-MDS, 8 males and 6 females, with a median age of 76 years, ranging from 66 to 88 years, that had been followed in the Hematology Department of Centro Hospitalar do Porto, Porto, Portugal, and that had at least one appointment at the hospital from September 2015 to November 2015.

An equal number of healthy controls (blood donors) were studied in parallel, 8 males and 6 females, with a median age of 55 years, ranging 19 to 63 years. First time donors, and donors with a history of infection in the previous 3 months and/or who have had neoplasms were excluded.

Clinical and laboratory data were retrospectively collected from the hospital records. Patients were considered to have anemia, neutropenia and thrombocytopenia if hemoglobin (Hg) < 12.5 g/dL, neutrophils < 2.000 × 10^6^/L and platelets < 150 × 10^9^/L, respectively.

The diagnosis and classification of MDS were established according to the WHO criteria, revised in 2016 [4, 5], after excluding other conditions that could potentially contribute to BM dysplasia and/or cytopenias. Only the following LR-MDS categories were included: MDS-SLD, MDS-MLD, MDS-RS and MDS-del(5q).

In order to avoid artefactual effects on neutrophil and monocyte immunophenotypes, patients who were being treated with granulocyte (G-CSF) or granulocyte-macrophage (GM-CSF) colony stimulating factors (CSF) at the time of the study or in the preceding 3 months were excluded, as did patients submitted to cytoreductive therapy, lenalidomide, hypomethylating and/or immunosuppressive treatments, and patients with concomitant infections or other neoplastic diseases. Previous or concomitant treatments with erythropoietin (EPO) and thrombopoietin receptor agonists were not exclusion criteria, neither did iron chelating therapies, vitamins or other nutrients.

The IPSS and the revised IPSS (IPSS-R) were calculated for all patients with MDS as previously described [6, 7]. Levels of Hg < 10.0 g/dL, neutrophils < 1800 × 10^6^/L and platelets < 100 × 10^9^/L were considered for risk stratification. The IPSS criteria were used to derive the karyotype-based risk classification [6].

Transfusion-related variables included the cumulative transfusion burden (total number of RBC units) and transfusion intensity (median number of RBC units/month). Transfusion-dependency was defined according to the WHO classification-based Prognostic Scoring System criteria [2].

Given the fact that elevated lactate dehydrogenase (LDH) has been associated with decreased overall survival [32], serum LDH levels were also evaluated. Moreover, as most patients with MDS were RBC transfusion-dependent [14, 33], and iron overload has been associated with worse prognosis in patients with LR-MDS [34], the serum ferritin levels were measured.

Bone marrow aspirate samples were used to prepare BM smears. These were stained with Leishman’s stain, and cell morphology was analyzed by conventional light microscopy. In each case, a Perls’ Prussian blue stain was performed. Other special stains were used whenever considered helpful. The acceptable quality of samples was defined according to the guidelines of the International Council for Standardization in Hematology [35]. Erythroid and granulocytic dysplasia were defined by the presence of ≥10% BM cells of the respective lineage with morphological alterations; presence of ≥15% ringed sideroblasts was also considered a diagnostic criteria for erythroid dysplasia. Megakaryocytic dysplasia was recognized by the presence of morphological abnormalities. Morphological features used for the definition of myeloblasts were those proposed by the International Working Group on Morphology of MDS [35], and the blast cell percentage was determined using the overall number of BM nucleated cells as denominator.

Bone marrow biopsy specimens were fixed in neutral-buffered formalin or Bouin’s fixative solution, decalcified, and embedded in paraffin-wax. Standard routine stains included hematoxylin & eosin and/or Giemsa, and Gömöri’s silver stain for the evaluation of BM fibrosis [36]. Immunohistochemistry was done in specific cases. The BM cellularity was estimated based on the age-adapted normal values [36], and dysmyelopoiesis was evaluated as previously described [37, 38].

Cytogenetics studies were performed in BM aspirates, either by conventional cytogenetics and/or fluorescence in situ hybridization (FISH). Conventional cytogenetics was performed on direct and 24- to 48-h unstimulated BM cultures, analyzed following trypsin Giemsa banding; 20 metaphases were evaluated. In FISH studies, VYSIS DNA FISH probes (Abbott Molecular Inc., IL, USA) were used to detect numeric and structural abnormalities on chromosomes 5, 7 and 20, and numeric abnormalities on chromosomes 8 and X. Two hundred interphase nuclei were counted. Cytogenetic classification was performed by grouping patients according to Schanz et al. [39].

### Flow cytometry studies

Peripheral blood samples were collected into ethylene-diamine-tetra-acetic acid tripotassium salt containing tubes and processed within 24 h after collection.

Cell immunophenotyping was performed by 8-color FCM using fluorochrome conjugated monoclonal antibodies (mAbs) with different specificities (Table 1). These mAbs were combined in two different tubes, conceived to quantify immature CD34^+^ cell in the PB and to evaluate cell surface antigen expression in circulating neutrophils (mostly tube 1) and monocytes (mostly tube 2) (Table 2). A normal PB sample was run in parallel with each patient PB sample.

**Table 1:** Specificities, clones, isotypes, fluorochromes and manufacturers of the monoclonal antibodies used in this study

Antigen	Clone	Isotype	Fluorochrome	Manufacturer
CD10	ALB1	IgG2a	PE-Cy7	BC-IOT
CD11b	D12	IgG2a	APC	BDB
CD11c	S-HCL-3	IgG2b	APC	BDB
CD13	L138	IgG1	PE	BDB
CD14	HCD14	IgG1	APC-H7	BL
CD15	MMA	IgM	FITC	BDB
CD16	3G8	IgG1	V450	BDH
CD34	8G12	IgG1	PerCP-Cy5.5	BDB
CD45	J.33	IgG1	KO	BC-IOT
CD56	N901-HLDA6	IgG1	PE-Cy7	BC-IOT
CD64	22	IgG1	PE	BC-IOT
HLA-DR	L243	IgG2a	FITC	BDB

Abbreviations: *APC* allophycocyanin, *Cy5.5* Cyanine 5.5, *Cy7* Cyanine 7, *FITC* Fluorescein, isothiocyanate, *KO* Khrome Orange, *PE* Phycoerythrin, *PerCP* Peridinin chlorophyll protein, *V450* Violet 450, *BC* Beckman Coulter, *BDB* Becton Dickinson Bioscience, *BDH* Becton Dickinson Horizon, *BL* Biolegend, *IOT* Immunotech

**Table 2:** Eight color-combinations of the fluorochrome-conjugated monoclonal antibodies used in this study

Tubes	FITC	PE	PerCP-Cy5	PE-Cy7	APC	APC-H7	V450	KO
1	CD15	CD13	CD34	CD10	CD11b	CD14	CD16	CD45
2	HLA-DR	CD64	CD34	CD56	CD11c	CD14	CD16	CD45

Abbreviations: *APC* allophycocyanin, *Cy5.5* Cyanine 5.5, *Cy7* Cyanine 7, *FITC* Fluorescein, isothiocyanate, *KO* Khrome Orange, *PE* Phycoerythrin, *PerCP* Peridinin chlorophyll protein, *V450* Violet 450

Cell staining was done using a whole blood stain-lyse-and-then-wash method, and the BD FACS™ Lysing Solution, according to the instructions of the manufacturer.

Sample acquisition was performed in a BD FACSCanto II™ flow cytometer. Forward scatter (FSC) and side scatter (SSC) were captured on a linear scale, and SSC was represented with a mathematical transformation (Exp-SSC-Low). For fluorescence parameters, a logarithmic amplification was used, with logical transformation. At least 200,000 cell events per tube were recorded and stored as flow cytometry standard (.fcs) 3.0 files.

Flow cytometer set-up and calibration was performed accordingly to the Euroflow consortium Standard Operating Procedures [40], available in [41]. Daily control was monitored by plotting fluorescence intensity values in Levy Jennings charts. External quality assessment/proficiency testing was performed by participating in the Euroflow Quality Assurance program [42].

**Fig. 1:**
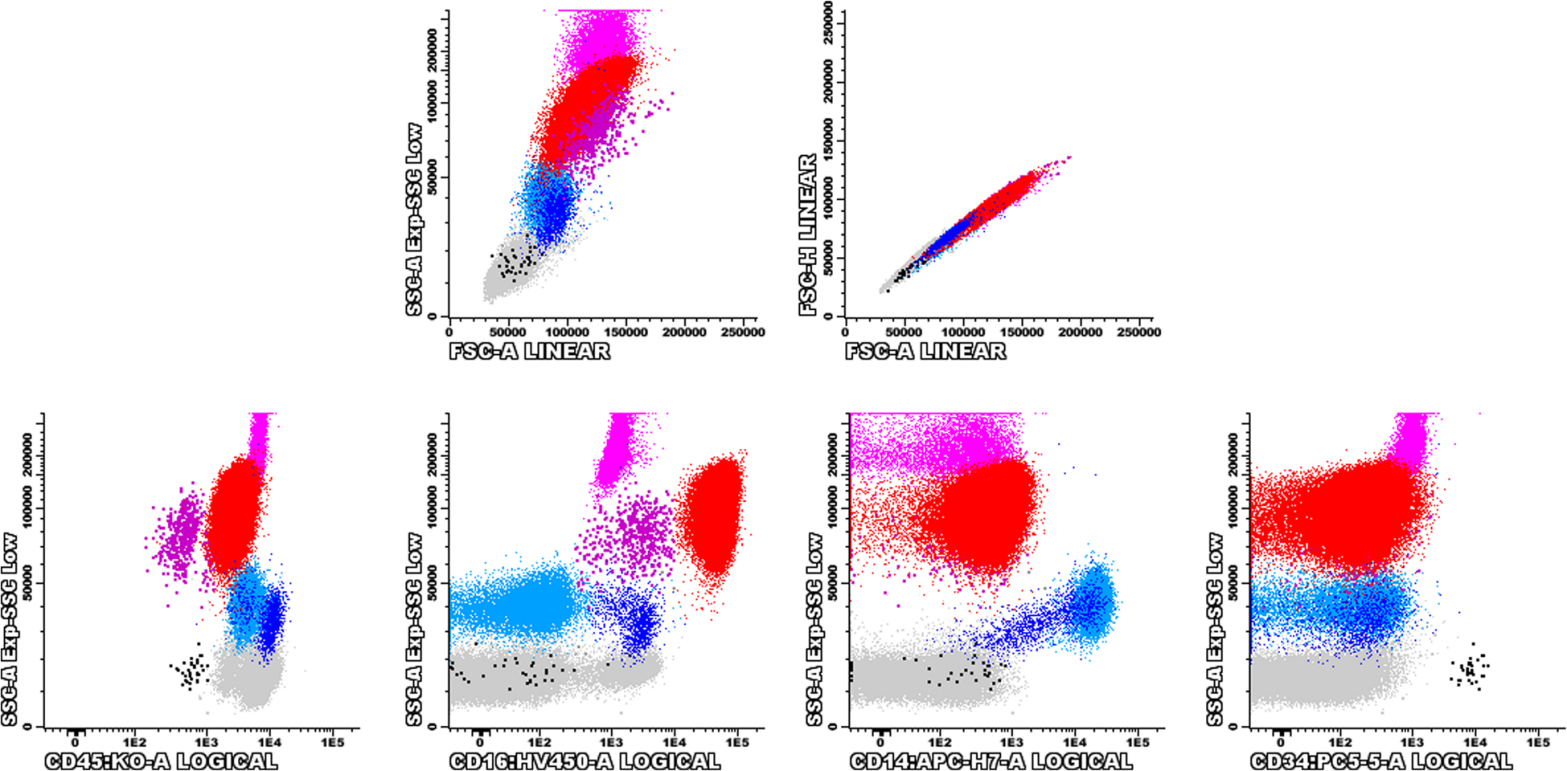
Bivariate dot-plots obtained from a normal PB sample, illustrating the analysis procedure used for the immunophenotypic identification, quantification and characterization of neutrophils, monocytes, and CD34^+^ cells, after excluding cell debris and doublets. Neutrophils (red dots; 61.16%) were selected based on their SSC/FSC characteristics and CD45^+low^CD16^+high^ expression. Monocytes (blue dots; 7.64%) were selected based on SSC/FSC and CD45^+int^CD14^+^ expression; subsequently, monocytes were separated into classical (light blue dots; 6.88%) and non-classical (either intermediate CD14^+high^CD16^+^ or proinflammatory CD14^+low^CD16^+^, dark blue dots; 0.76%) monocyte subsets. Immature CD34+ cells (black dots, 0.02%) were identified based on SSC/FSC and CD45^+low^CD34^+^ expression. Other cells: eosinophils (pink dots; 4.93%); immature granulocytes, including promyelocytes, myelocytes and metamyelocytes (purple dots; 0.27%); lymphocytes and basophils (gray dots; 25.97%). Abbreviations: FSC, forward scatter; PB, peripheral blood; SSC, side scatter

Flow cytometry data analysis was done using the Infinicyt™ software (Cytognos, Salamanca, Spain). Neutrophils, monocytes and blast cells were identified and classified according to their SSC and FSC characteristics and antigen expression profiles, as described in Fig. 1. Monocytes were further subdivided into classical (CD14^+high^CD16^−^), intermediate (CD14^+high^CD16^+^) and proinflammatory (CD14^+low^CD16^+^) monocytes. Non-classical (CD14^+^CD16^+^, intermediate + proinflammatory) monocyte subsets were considered together in succeeding analysis. Neutrophils and monocytes were analyzed for the levels of expression of CD10, CD11b, CD11c, CD13, CD15, CD16, CD56, CD64, and HLA-DR, by recording the median fluorescence intensity (MedFI) for each marker. The relative representation of CD14^+^CD16^−^ and CD14^+^CD16^+^ monocyte subsets and the percentage of CD56^+^ monocytes were also recorded.

The FSC/SSC values and MedFI values obtained in neutrophils and monocytes from patients with LR-MDS were considered abnormal when they were out of the mean value ±2 standard deviations (SD) of the same parameter obtained in PB neutrophils and monocytes from healthy individuals.

### Immunophenotypic scores for myelodysplasia

#### Neutrophil scores

Two different scoring schemas were used for neutrophils. The scoring schema type 1 was based only on the number of abnormally low parameters found in PB neutrophils (below the mean-2SD of the values found for the same parameter in normal PB neutrophils). The scoring schema type 2 took into account both the number of abnormally low parameters and the severity of each parameter deficiency, as previously described [11, 23].

In both cases, values within the mean ± 2SD or above the mean + 2SD were scored with “0”, and in both cases only 8 of the parameters analyzed (FSC, SSC, CD10, CD11b, CD11c, CD13, CD16 and CD45) were considered for scoring. The neutrophil score, ranging from 0 to 8 in both cases, was calculated for each patient by adding up the scores obtained for each of the 8 parameters evaluated. Three groups corresponding to an overall score of “0”, 1 and ≥2 were arbitrarily considered equivalent to “no neutrophil dysplasia”, “possible neutrophil dysplasia” and “neutrophil dysplasia”, respectively. Cases with a neutrophil score ≥ 2 were subsequently arbitrarily classified as having mild (scores 2 and 3), moderate (scores 4, 5 and 6) and severe (scores 7 and 8) neutrophil dysplasia, respectively.

##### Neutrophil scoring schema 1

Each abnormally low parameter (below the mean-2SD) was scored with 1 point.

##### Neutrophil scoring schema 2

Values of 0.25, 0.5 or 1.0 were given when the MedFI value obtained for each of the phenotypic variables evaluated were between the mean-2SD and the mean-3SD, between the mean-3SD and the mean-4 SD, or below the mean-4SD of the values found for the same parameter in normal PB neutrophils, respectively.

#### Monocyte scores

For monocytes, only the percentages of CD14^+^CD56^+^ and CD14^+^CD16^+^ monocytes on total monocytes were used to calculate the FCM monocyte score for myelodysplasia. Aberrant high CD56 expression was defined as a percentage of CD56^+^ cells exceeding the mean + 2SD of the values found in normal PB monocytes. Abnormal low CD16 expression was defined as a percentage of CD14^+^CD16^+^ monocytes of less than 5% of total monocytes. Depletion of non-classical monocytes was arbitrarily considered mild, moderate or severe when CD14^+^CD16^+^ cells accounted for less than 5%, 2.5% and 1.25% of total monocytes, respectively. The overall monocyte score, ranging from 0 to 2, was calculated for each patient by adding up the scores obtained for each of the 2 parameters evaluated.

As described for neutrophils, two different scoring schemas were used for monocytes: scoring schema type 1, based only on the number abnormal parameters found in PB monocytes, and scoring schema type 2, taking into account both the number of abnormal parameters and the severity of each parameter abnormality. The monocyte score, ranging from 0 to 2, was calculated for each patient by adding up the scores obtained for each of the 2 parameters evaluated. Three groups corresponding to an overall score of 0, 1 and 2, were arbitrarily considered equivalent to “no monocyte dysplasia”, “possible monocyte dysplasia” and “monocyte dysplasia”, respectively.

##### Monocyte scoring schema 1

Each abnormal parameter (percentage of CD56^+^ cells exceeding the mean + 2SD and percentage of CD16^+^ cells < 5%) was scored with 1 point.

##### Monocyte scoring system 2

Values of 0.25, 0.5 or 1 were given when the % of CD56^+^ monocytes were between the mean + 2SD and the mean + 3SD, between the mean + 3SD and the mean + 4 SD, or above the mean + 4SD of the values found for the same parameter among normal PB monocytes, respectively. Values of 0.25, 0.5 or 1 were assumed when the percentages of CD16^+^ monocytes were between the 2.5 and 5.0%, between 1.25% and 2.5% and below 1.25%, respectively.

#### Myeloid immunophenotypic scores

Two myeloid immunophenotypic MDS scores, type 1 and type 2, both ranging from 0 to 10, were obtained for each patient by adding up the correspondent neutrophil and monocytic MDS scores. Three groups corresponding to overall scores of “0”, 1 and ≥2 were arbitrarily considered equivalent to “no myelodysplasia”, “possible myelodysplasia” and “myelodysplasia”, respectively. Cases with a myeloid score ≥ 2 were subsequently classified as having mild (scores 2 to 4), moderate (scores 5 to 7) and severe (scores 8 to 10) myelodysplasia, respectively.

### MDS thermometer

The MDS Thermometer was conceived as a screening tool for detection and monitoring of MDS in clinical practice. It consists in a visual analogue scale rated from 0 to 10 points (myeloid thermometer) with two domains (neutrophil thermometer, rated from 0 to 8, and monocyte thermometer, rated from 0 to 2) based on the immunophenotypic scores defined above. For simplicity, only the neutrophil and monocyte scoring schemas type 1 were used to construct the MDS Thermometer presented in this paper. The concept was based on the Emotion Thermometers Tool, created by Alex J Mitchell [43, 44].

### Statistical analysis

Results were expressed as absolute and relative frequencies, or as mean, median, SD, minimum and maximum values. Results obtained in PB samples of MDS patients were compared with those obtained in control PB samples. The non-parametric Mann-Whitney U test was used to compare the MedFI observed for each marker in PB neutrophils and monocytes from MDS patients and controls, as well as to compare blood cell counts at the time of the study with those at diagnosis. *P* values < 0.05 were considered statistically significant. All statistical analyses were performed using the SOFA Statistics version 1.4.5 (Paton-Simpson & Associates Ltd., Auckland, New Zealand).

## Results

### Study population

Table 3 summarizes the demographic, clinical and laboratory data of the study population. Detailed data can be found in Supplementary Material (Additional file 1: Table S1).

**Table 3:** MDS categories and risk stratification of the patients included in this study

Diagnosis (WHO 2016)^a^	
MDS-RS	6/14 (43%)
MDS-SLD	4/14 (29%)
MDS-MLD	3/14 (21%)
MDS-del(5q)	1/14 (7%)
International Prognostic Scoring System (IPSS)	
IPSS score	0.3 ± 0.4; 0 (0–1.0)
Score = 0.0	8/14 (57%)
Score = 0.5	3/14 (21%)
Score = 1.0	3/14 (21%)
Score > 1.0	0/14 (0%)
IPSS risk groups	
Low risk	8/14 (57%)
Intermediate risk 1	6/14 (43%)
Intermediate risk 2	0/14 (0%)
High risk	0/14 (0%)
Cytogenetic based risk classification^b^	
Very good	0/10 (0%)
Good	9/10 (90%)
Intermediate	1/10 (10%)
Poor	0/10 (0%)
Very poor	0/10 (0%)

Abbreviations: *IPSS* International Prognostic Scoring System, *MDS* myelodysplastic syndromes, *MDS-del(5q)* MDS with isolated deletion of chromosome 5q, *MDS-MLD* MDS with multilineage dysplasia, *MDS-RS* MDS with ring sideroblasts, *MDS-SLD* MDS with single lineage dysplasia, *WHO* World Health Organization

Results are expressed as absolute and (relative) frequencies

^a^ Patients with MDS unclassifiable or with ≥20% blast in the BM were omitted, as did patients with MDS with excess of blasts, and patients diagnosed with Myelodysplastic/myeloproliferative neoplasms

^b^ Cytogenetic scoring: Very good: -Y, del(11q); Good: normal, del(5q), del(12p), del(20q), double including del(5q); Intermediate: del(7q), + 8, + 19, i(17q), any other single or double independent clones; Poor: −7, inv.(3)/t(3q), del3q, double including − 7/del(7q), complex (3 abnormalities); Very poor: complex (> 3 abnormalities)

According to the WHO criteria, 6 patients were classified as MDS-RS, 4 patients had MDS-SLD, 3 patients had MDS-MLD, and 1 patient was classified as MDS-isolated 5q- (Table 3).

Using the IPSS, all patients were categorized as LR-MDS: 8 patients had low IPSS risk and 6 patients had intermediate 1 IPSS risk (Table 3).

Cytogenetic based stratification revealed good or intermediate risk in 9 cases and 1 case, respectively (Table 3). Overall, metaphase cytogenetics and/or interphase FISH testing for − 5/5q-, − 7/7q-, + 8, and 20q-, identified in cytogenetic aberrancies in only 2 out 10 cases (20%), corresponding to isolated del(5q) (1 patient) and isolated monosomy 8 (1 case); cytogenetic data were unavailable in 4 cases (Additional file 1: Table S1).

The median time from the diagnosis was 7.6 years, ranging from 0.5 to 12.6 years.

At diagnosis, all the MDS patients had anemia, but only 5 (36%) had neutropenia and only 4 (29%) had thrombocytopenia (Additional file 1: Table S1). The median values of the Hg levels, and neutrophil and platelet counts were of 9.1 g/dl, 2567 × 10^6^/L, and 200 × 10^9^/L ranging from 7.2 to 11.9, 575 to 7025 and 61 to 591, respectively. Abnormal RBC morphology in the PB smears were present in all cases, whereas abnormal neutrophil and/or platelet morphological features were observed in 4 patients (29%) each. At the time of the study, the Hg levels were significantly lower than those observed at the diagnosis (*p* = 0.006), despite of RBC transfusions; no statistically significant differences were found for the neutrophil and platelet counts, neither for the percentage of blasts in the PB (*p* > 0.05). In addition, although a higher fraction of patients had neutropenia and/or thrombocytopenia, as compared to that observed at diagnosis, differences were also not statistically significant (p > 0.05) (Additional file 1: Table S1 footnote).

Most patients had received RBC transfusions, and most of them had been treated with hematopoietic growth factors at some point during the course of the disease (Additional file 1: Table S1). Concerning blood transfusions, 13 patients (93%) had received at least one RBC unit, and eleven (79%) had been regularly transfused (transfusion-dependent). The median number of RBC units received per transfused patient was of 57, ranging from 15 to 462, and a median number of RBC transfused per patient/month was of 1.4, ranging from 0.3 to 4.0 (Additional file 1: Table S1). The mean ferritin serum levels were of 1854 ± 1126 ng/ml, with 93% of the patients showing increased serum ferritin values, compatible with iron overload. None of the patients was receiving iron-chelating therapies. In addition, none was medicated with myeloid growth factors (exclusion criteria), although 5 patients had been treated with G-CSF in the past. Only 2 patients were being treated with EPO at the time of the study, although most of them had previously received EPO. No patients had ever received GM-CSF or thrombopoietin receptor agonists. Increased serum LDH levels were seen in only 3 cases (23%), at the time of the study (Additional file 1: Table S1).

### Flow cytometry studies

#### Peripheral blood neutrophils

**Fig. 2:**
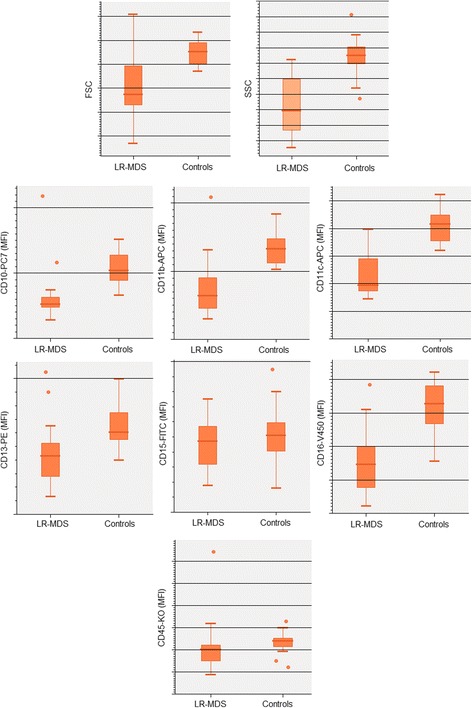
FSC, SSC and surface antigen expression in PB neutrophils from patients with LR-MDS and healthy individuals (controls). Results as expressed as arbitrary units of fluorescence intensity. Lower whiskers are 1.5 times the Inter-Quartile Range below the lower quartile, or the minimum value, whichever is closest to the middle. Upper whiskers are calculated using the same approach. Outliers are displayed. Abbreviations: FSC, forward scatter; LR-MDS, lower risk myelodysplastic syndromes; MFI, median fluorescence intensity; PB, peripheral blood; SSC, side scatter

**Fig. 3:**
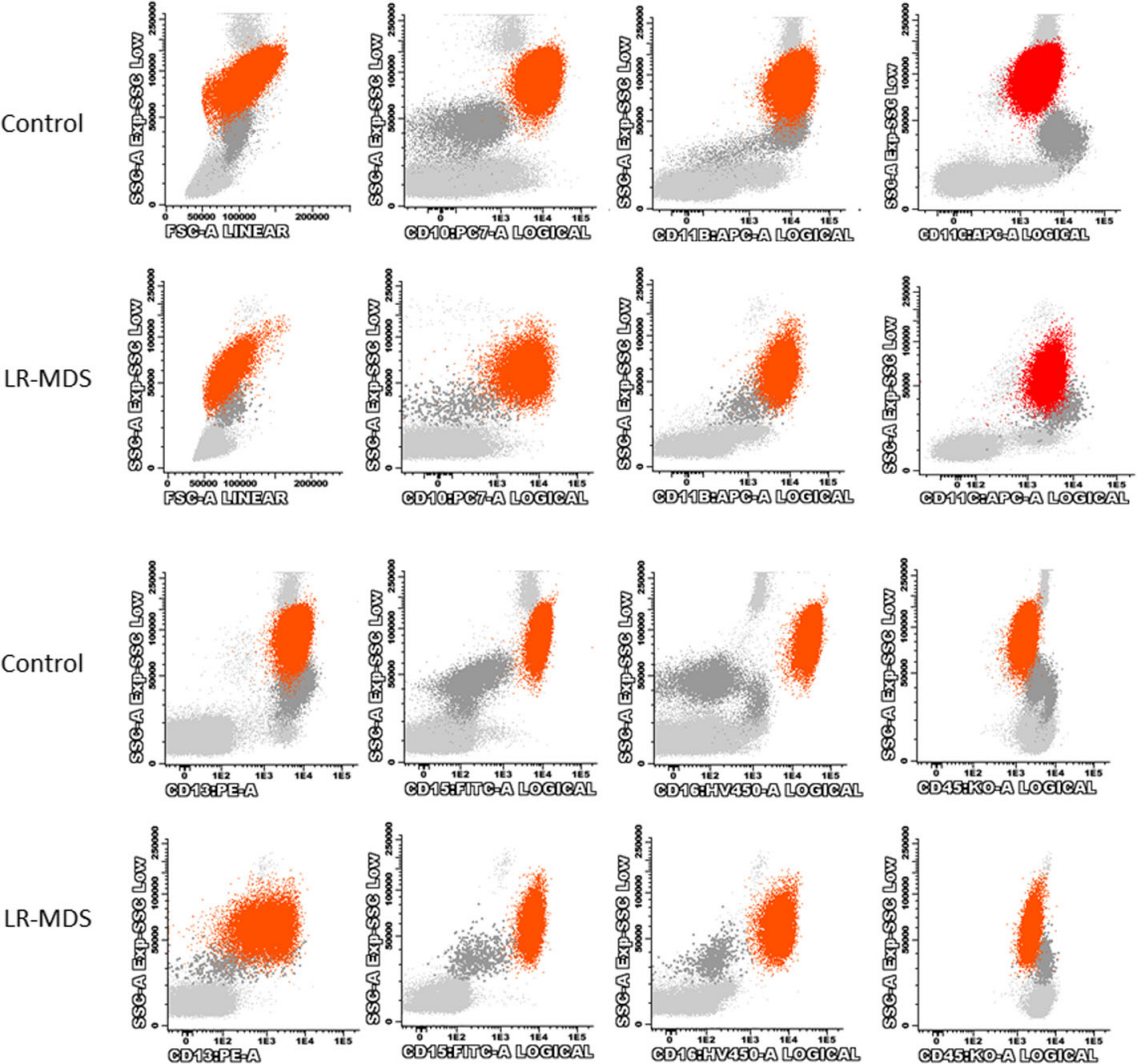
Bivariate SSC/FSC, SSC/CD10, SSC/CD11b, SSC/CD11c, SSC/CD13, SSC/CD15, SSC/CD16 and SSC/CD45 dot plots illustrating the decreased FSC/SSC and the diminished expression of cell surface molecules (CD10, CD11b, CD13 and CD16) in the PB neutrophils (red dots) from one patient with LR-MDS, as compared to a healthy individual (control). Red dots, neutrophils; light gray dots, lymphocytes and eosinophils; dark gray dots, monocytes. Abbreviations: FSC, forward scatter; LR-MDS, lower risk myelodysplastic syndromes; PB, peripheral blood; SSC, side scatter

Neutrophils from patients with LR-MDS had lower FSC and SSC values as compared with controls (*p* = 0.008 and *p* < 0.001, respectively) (Figs. 2 and 3, and Additional file 2: Table S2).

In addition, neutrophils from patients with LR-MDS had significantly decreased expression of CD10 (*p* < 0.001), CD11b (*p* < 0.001), CD11c (*p* < 0.001), CD13 (*p* = 0.022) and CD16 (*p* = 0.002) when compared to normal individuals. No statistically significant differences were observed for CD15 and CD45 expression (*p* > 0.05), although both markers had a slight reduction in fluorescence intensity in patients, as compared to controls.

When analyzed individually, neutrophils from most LR-MDS patients had abnormally low FSC and/or SSC (71% and 57% of cases, respectively) (Table 4). Among the cell surface molecules evaluated, CD11b, CD11c, CD10, CD16, CD13 and CD45 were the most frequently affected, in that order; the percentage of cases showing reduced levels of expression of these molecules were of 71%, 71%, 64%, 43%, 29% and 14%, respectively (Table 4). Overall, 13 patients (93%) had abnormally low expression of 2 or more (out of 9) molecules on neutrophils, with 7 patients (50%) having abnormally low expression of 5 or more parameters. In addition, 4 cases had abnormally high expression of CD10, CD11b, CD13 or CD45 on circulating neutrophils (1 case each).

**Table 4:** Type and frequency of immunophenotypic aberrancies detected in PB neutrophils from LR-MDS patients versus normal individuals (controls**)**

	LR-MDS (n = 14)		Controls (*n* = 14)	
	Decreased	Increased	Decreased	Increased
Abnormal light scatter characteristics				
FSC	10 (71%)	1 (7%)	0 (0%)	0 (0%)
SSC	8 (57%)	0 (0%)	0 (0%)	1 (7%)
Abnormal expression of cell surface markers				
CD10	9 (64%)	1 (7%)	0 (0%)	0 (0%)
CD11b	10 (71%)	1 (7%)	0 (0%)	1 (7%)
CD11c	10 (71%)	0 (0%)	0 (0%)	0 (0%)
CD13	4 (29%)	1 (7%)	0 (0%)	0 (0%)
CD15	0 (0%)	0 (0%)	1 (7%)	1 (7%)
CD16	6 (43%)	0 (0%)	0 (0%)	0 (0%)
CD45	2 (14%)	1 (7%)	1 (7%)	0 (0%)
Number of individuals with abnormal parameters				
No abnormal parameters	1 (7%)	10 (71%)	12 (86%)	11 (79%)
One abnormal parameter	0 (0%)	3 (21%)	2 (14%)	3 (21%)
Two abnormal parameters	2 (14%)	1 (7%)	0 (0%)	0 (0%)
Three abnormal parameters	1 (7%)	0 (0%)	0 (0%)	0 (0%)
Four abnormal parameters	3 (21%)	0 (0%)	0 (0%)	0 (0%)
Five abnormal parameters	4 (29%)	0 (0%)	0 (0%)	0 (0%)
Six abnormal parameters	2 (14%)	0 (0%)	0 (0%)	0 (0%)
Seven abnormal parameters	0 (0%)	0 (0%)	0 (0%)	0 (0%)
Eight abnormal parameters	1 (7%)	0 (0%)	0 (0%)	0 (0%)
Nine abnormal parameters	0 (0%)	0 (0%)	0 (0%)	0 (0%)

Abbreviations: *LR-MDS* lower risk myelodysplastic syndromes, *PB* peripheral blood

Results are expressed as absolute and (relative) frequencies

Percentages were approximated to the closest full unit

In contrast, abnormal levels of cell surface molecules were found in only 4 controls, and the phenotypic abnormalities were restricted to one parameter in all cases (abnormally high CD11b levels: *n* = 1; abnormally high CD15 levels: n = 1; abnormally low CD15 levels: n = 1; abnormally low CD45 levels: n = 1).

The only patient with a normal neutrophil immunophenotype was a 75 year-old female classified as having MDS-SLD, who had mild macrocytic anemia (Hg 9.0 g/dL, MCV 104.1 fL) and mild thrombocytopenia (platelet count of 100 × 10^6^/L). The BM was hypocellular, with 5.3% blast cells, increased myeloid/erythroid ratio (3.9) and mild dyserythropoiesis, with normal myeloid and megakaryocytic lineages. BM cytogenetics have revealed a 46,XX karyotype with 3 metaphases with non-clonal aneuploidies. The time from the diagnosis was of 31 months, the patient had been occasionally transfused (total of 3 RBC units), and the cytopenias have remained stable over time.

When the healthy controls were separated in two groups according to age (< 55 years old, *n* = 7 versus ≥ 55 years old, n = 7), we did not observe statistically significant differences for any of the parameters analyzed on neutrophils; also, no differences were found between males and females (*p* > 0.05 in all cases).

#### Peripheral blood monocytes

**Fig. 4:**
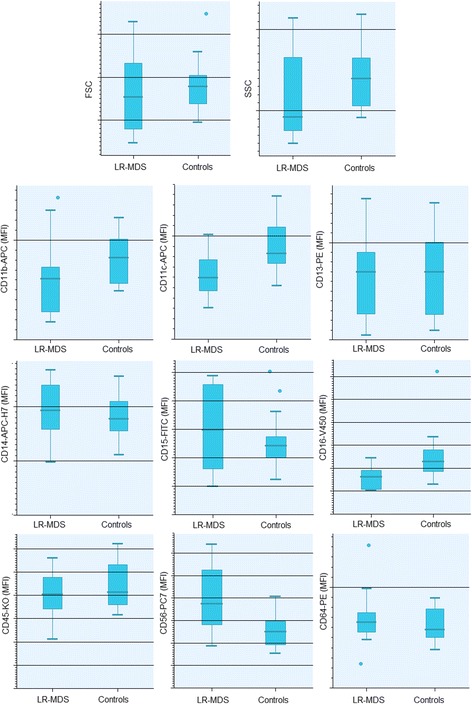
FSC, SSC and surface antigen expression in PB monocytes from patients with LR-MDS and healthy individuals (controls). Results as expressed as arbitrary units of fluorescence intensity. Lower whiskers are 1.5 times the Inter-Quartile Range below the lower quartile, or the minimum value, whichever is closest to the middle. Upper whiskers are calculated using the same approach. Outliers are displayed. Abbreviations: FSC, forward scatter; LR-MDS, lower risk myelodysplastic syndromes; MFI, median fluorescence intensity; PB, peripheral blood; SSC, side scatter

In general, monocytes from patients with LR-MDS had light scatter properties similar to the monocytes from normal individuals (p > 0.05) (Fig. 4 and Additional file 3: Table S3). In the same way, the overall levels of CD13, CD14, CD15, CD45 and CD64 expression on monocytes did not differ significantly from those observed in controls (p > 0.05). However, monocytes from patients with LR-MDS had significantly higher levels of CD56 (*p* = 0.006), and lower levels of CD11c (*p* = 0.004), CD16 (*p* = 0.005) and HLA-DR (*p* = 0.042), and showed a tendency for a lower CD11b expression (*p* = 0.089), as compared to controls.

Despite of the above-mentioned differences between monocytes from LR-MDS patients and controls, the individual analysis of the analyzed cell surface markers on monocytes was much less informative than that found in neutrophils, with an abnormal monocyte immunophenotype being found in only a limited number of cases.

**Fig. 5:**
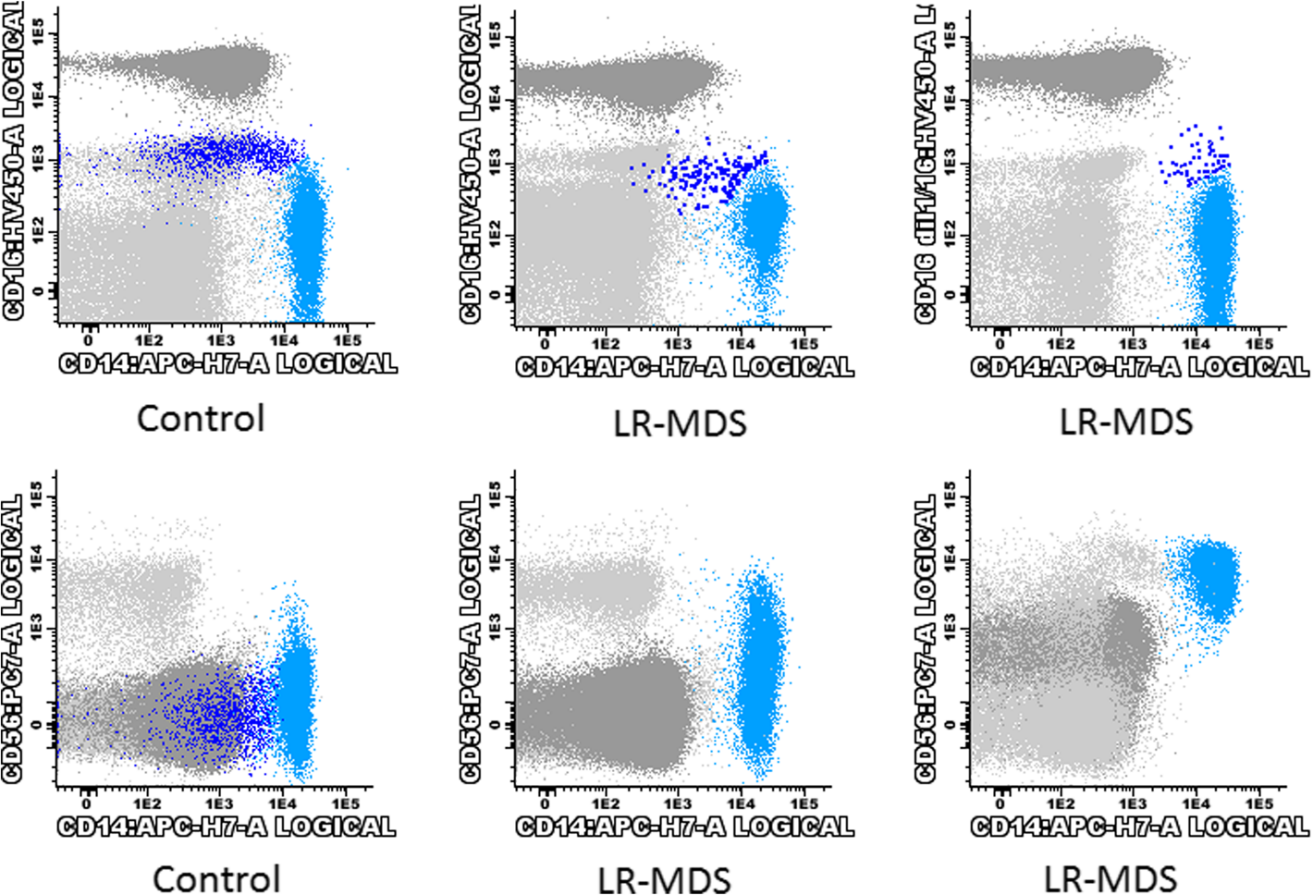
Bivariate CD16/CD14 and CD56/CD14 dot plots illustrating the depletion of non-classical (intermediate CD14^+high^CD16^+^ plus proinflammatory CD14^+low^CD16^+^) monocytes (upper row) and the aberrant CD56 expression in the PB classical CD14^+high^CD16^−^ monocytes (lower row) from two patients with LR-MDS, as compared to a normal individual (control). Light blue dots, classical CD14^+high^CD16^−^ monocytes; dark blue dots, non-classical (intermediate CD14^+high^CD16^+^ plus proinflammatory CD14^+low^CD16^+^) monocytes; light gray dots, lymphocytes and eosinophils; dark gray dots, neutrophils. Abbreviations: FSC, forward scatter; LR-MDS, lower risk myelodysplastic syndromes; PB, peripheral blood; SSC, side scatter

One of the most consistent aberrancies found in MDS monocytes consisted in abnormally high levels of CD56 expression, observed in 6 cases (43%), with increased percentages of CD56^+^ monocytes. In accordance, monocytes from LR-MDS patients had significantly higher percentages of CD56^+^ cells, as compared to normal individuals (median values of 15% and 7%, ranging from 0 to 99% and from 0 to 15%, respectively; *p* = 0.026) (Fig. 5 and Table 5). Increased percentages of CD14^+^CD56^+^ monocytes (> 18%) were found in 6 (43%) MDS patients but in none of the healthy individuals, and in most of the patients (4, 29%), CD14^+^CD56^+^ monocytes were markedly increased (> 28%). CD56 expression was mainly observed on classical CD14^+high^CD16^−^ monocytes (Fig. 5, panel B).

**Table 5:** Immunophenotypic alterations detected in PB monocytes from LR-MDS patients versus normal individuals (controls)

	LR-MDS (n = 14)	Controls (n = 14)
Increased CD56 expression in monocytes		
CD14^+^CD56^+^ cells (% total CD14^+^ monocytes) (*)	15 (0–99)	7 (0–15)
Increased percentages of CD14^+^ 56^+^ cells (> 18.0% total CD14^+^ monocytes)	6 (43%)	0 (0%)
Severity		
Mild: CD56^+^ cells]18%–23%]	1 (7%)	0 (0%)
Moderate: CD56^+^ cells [23%–28%]	1 (7%)	0 (0%)
Severe: CD56^+^ cells]28%–100%]	4 (29%)	0 (0%)
Decreased CD16 expression in monocytes		
CD14^+^CD16^+^ cells (% total CD14^+^ monocytes) (**)	3 (0–8)	12 (4–28)
Decreased percentages of CD14^+^CD16^+^ cells (< 5.0% of total CD14^+^ monocytes)	9 (64%)	2 (14%)
Severity		
Mild: CD16^+^ cells [2.5%–5.0%]	2 (14%)	2 (14%)
Moderate: CD16^+^ cells [1.25–2.5%]	1 (7%)	0 (0%)
Severe: CD16^+^ cells [0.00–1.25%]	6 (43%)	0 (0%)

Abbreviations: *LR-MDS* lower risk myelodysplastic syndromes, *PB* peripheral blood

Results are expressed as absolute and (relative) frequencies or as median (range) values

Percentages were approximated to the closest full unit

Mann-Whitney U test, LR-MDS patients versus controls: (*) P = 0.026; (**) P < 0.001

Another recurrent aberrancy observed in LR-MDS patients consisted in a marked decrease in the fraction of CD14^+^CD16^+^ monocytes, comparatively to controls (median values of 3% and 12%, ranging from 0 to 8% and from 4 to 28%, in patients and in controls, respectively; *p* < 0.001) (Fig. 5 and Table 5). In overall, 9 patients (64%) had decreased percentages of CD14^+^CD16^+^ monocytes (< 5%), when compared to only 2 (14%) of the controls. Curiously, the severity of the deficiency was higher in patients than in controls, with 50% of the patients, but none of the controls, having a moderate (< 2.5%) or severe (< 1.25%) deficiencies of CD14^+^CD16^+^ monocytes.

Finally, abnormally low CD11b and HLA-DR expression was found in 5 (36%) and 4 (28%) patients, respectively. Less frequent findings included abnormally low levels of CD45 (2 patients, 14%), and decreased CD11c and CD64 expression (1 case each, 7%); moreover, abnormally high levels of CD14 and CD64 were found in 2 cases each, and abnormally high levels of CD11b in only 1 case; cases with abnormal CD13 expression were not found.

When the healthy volunteers aged 55 years or over were compared to those who were younger than 55 years, we observed a tendency for a lower intensity of CD14 expression in monocytes (*p* = 0.064), and a higher percentage of proinflammatory (CD16^+^) monocytes in the first group (median values of 13.2% and 5.9%, respectively; *p* = 0.035). The other parameters analyzed did not show statistically significant differences between the “youngest” and “oldest” individuals, neither between males and females (*p* > 0.05).

#### Circulating immature cells

Immature CD34^+^ cells represented 0.15 ± 0.28% of the WBC in the PB from LR-MDS patients (median value of 0.05%, ranging from 0.01 to 1.05%), as compared to 0.03 ± 0.01% (median value of 0.03%, ranging from 0.01 to 0.04%) in normal individuals. These cells had low SSC and FSC, they were CD45^+low^, CD34^+^, CD13^+^, CD117^−/+^, HLA-DR^+^, and they virtually fail to express all the other molecules analyzed (CD10, CD11b, CD11c, CD15 and CD16) (data not shown).

Patients with MDS also had increased numbers of circulating immature granulocytes (promyelocytes, myelocytes and metamyelocytes) (1.17 ± 2.03%, with a median value of 0.36%, ranging from 0.06 to 7.72%), as compared to controls (0.11 ± 0.06%; median value of 0.11%, ranging from 0.02 to 0.21%). These cells were SSC^high^, FSC^high^, CD45^+low^, CD15^+^ and CD64^+^, and they had variable and low CD11b and CD16 expression, being negative for the remaining molecules tested (CD10, CD11c, CD13, CD34, CD56, CD117, HLA-DR) (data not shown).

Differences between patients and controls reached statistical significance for circulating immature granulocytes (*p* < 0.001), but not for immature CD34^+^ cells (*p* = 0.056).

### Immunophenotypic scores

As mentioned above, we defined two FCM-based scorings systems (type 1 and 2) to evaluate dysplasia in neutrophils and monocytes, and, for each scoring system, a total myeloid score was calculated. The results obtained are summarized in Tables 6 and 7, respectively.

**Table 6:** Neutrophil and monocyte immunophenotypic scores, and total myeloid score for myelodysplasia in the PB from LR-MDS patients versus normal individuals (controls), taking in account the number of phenotypic abnormalities found in each case

	LR-MDS (n = 14)	Controls (*n* = 14)
Neutrophil immunophenotypic score 1 (0–8)		
Score	5 (0–8)	0 (0–1)
Score rank (Σ)	59	1
Score classes		
0 (no dysplasia)	1 (7%)	13 (93%)
1 (possible dysplasia)	0 (0%)	1 (7%)
2–3 (mild dysplasia)	3 (21%)	0 (0%)
4–6 (moderate dysplasia)	9 (64%)	0 (0%)
7–8 (severe dysplasia)	1 (7%)	0 (0%)
Monocyte immunophenotypic score 1 (0–2)		
Score	1 (0–2)	0 (0–1)
Score rank (Σ)	15	2
Score classes		
0 (no dysplasia)	2 (14%)	12 (86%)
1 (possible dysplasia)	8 (57%)	2 (14%)
2 (dysplasia)	4 (29%)	0 (0%)
Myeloid immunophenotypic score 1 (0–10)		
Score	6 (0–8)	0 (0–1)
Score rank (Σ)	74	3
Score classes		
0 (no dysplasia)	1 (7%)	11 (79%)
1 (possible dysplasia)	0 (0%)	3 (21%)
2–4 (mild dysplasia)	3 (21%)	0 (0%)
5–7 (moderate dysplasia)	7 (50%)	0 (0%)
8–10 (severe dysplasia)	3 (21%)	0 (0%)

Abbreviations: *LR-MDS* lower risk myelodysplastic syndromes, *PB* peripheral blood

Results are expressed as absolute and (relative) frequencies or as median (range) values

Percentages were approximated to the closest full unit

Neutrophil score: The following parameters were considered: FSC, SSC, CD10, CD11b, CD11c, CD13, CD16, and CD45. Each abnormally low parameter (< mean – 2D of the values observed in controls) was scored with 1 point. Values within the mean ± SD or above the mean + 2SD were scored with “0”

Monocyte score: The following parameters were considered: % of CD16+ monocytes and % of CD56+ monocytes. These parameters were scored as follows: CD14 + CD56+ monocytes: ≤18% (0 points); > 18% (1 point); CD14 + CD16+ monocytes: ≥5% (0 points); < 5% (1 point)

Myeloid score: obtained by adding up the neutrophil and the monocytic score achieved for each patient

The following score rankings were obtained for each of the parameters analyzed: Patients: FSC = 10; CD11b = 10; CD11c = 10; CD10 = 9; SSC = 8; CD13 = 4; CD15 = 0; CD16 = 6; CD45 = 2. Controls: CD15 = 1; CD45 = 1; other parameters = 0

**Table 7:** Neutrophil and monocyte immunophenotypic scores, and total myeloid score for myelodysplasia in the PB from LR-MDS patients versus normal individuals (controls), taking in account the number and the severity of the phenotypic abnormalities found in each case

	LR-MDS (n = 14)	Controls (n = 14)
Neutrophil immunophenotypic score 2 (0–8)		
Score	2 (0–3)	0 (0–0)
Score rank (Σ)	28	0
Score classes		
0 (no dysplasia)	1 (7%)	14 (100%)
1 (possible dysplasia)	2 (14%)	0 (0%)
2–3 (mild dysplasia)	11 (79%)	0 (0%)
4–6 (moderate dysplasia)	0 (0%)	0 (0%)
7–8 (severe dysplasia)	0 (0%)	0 (0%)
Monocyte immunophenotypic score 2 (0–2)		
Score	1 (0–2)	0 (0–0)
Score rank (Σ)	12	1
Score classes		
0 (no dysplasia)	5 (36%)	14 (100%)
1 (probable dysplasia)	6 (43%)	0 (0%)
2 (dysplasia)	3 (21%)	0 (0%)
Myeloid immunophenotypic score 2 (0–10)		
Score	3 (0–5)	0 (0–1)
Score rank (Σ)	39	1
Score classes		
0 (no dysplasia)	1 (7%)	14 (0%)
1 (possible dysplasia)	1 (7%)	0 (0%)
2–4 (mild dysplasia)	11 (79%)	0 (0%)
5–7 (moderate dysplasia)	1 (7%)	0 (0%)
8–10 (severe dysplasia)	0 (0%)	0 (0%)

Abbreviations: *LR-MDS* lower risk myelodysplastic syndromes, *PB* peripheral blood

Results are expressed as absolute and (relative) frequencies or as median (range) values

Percentages were approximated to the closest full unit

Neutrophil score: The following parameters were considered: FSC, SSC, CD10, CD11b, CD11c, CD13, CD16, and CD45. Values within the mean ± SD or above the mean + 2SD were scored with “0”. Abnormally low parameter (< mean-2D of the values observed in controls) were scored as follows: (< mean-2D: 0.25 points; < mean-3SD: 0.5 points; < mean-3SD: 1 point

Monocyte score: The following parameters were considered: % of CD16+ monocytes and % of CD56+ monocytes. These parameters were scored as follows: CD14 + CD56+ monocytes: ≤18% (0 points); >mean + 2SD (18%): 0.25 points; > mean + 3SD (23%): 0.5 points; > mean + 4SD (28%): 1 point. CD14 + CD16+ monocytes: ≥5%: 0 points; CD14 + CD16+ monocytes < 5%: 0.25 point; CD14 + CD16+ monocytes: < 2.5%: 0.5 points; CD14 + CD16+ monocytes: < 1.25%: 1 point

Myeloid score: obtained by adding up the neutrophil score 1 and the monocytic score achieved for each patient

The following score rankings were obtained for each of the parameters analyzed: Patients: FSC = 6.5; CD11c = 5.5; CD11b = 5.0; SSC = 4.5; CD10 = 2.5; CD16 = 2; CD13 = 1.0; CD15 = 0.0; CD45 = 0.5. Controls: CD15 = 0.25; CD45 = 0.25; other parameters = 0

### Immunophenotypic scoring schema 1

#### Neutrophil score type 1

The mean neutrophil score type 1 obtained in LR-MDS patients was of 4 ± 2, ranging from “0” to 8 (total score rank = 59), while all controls had a score of 1 or lower (total score rank = 1), with only 1 case having a score = 1, corresponding to a normal individual whose neutrophils had dimmer CD45 expression (Table 6). According to this score, four groups of MDS patients were identified: score of “0”, no neutrophil dysplasia (*n* = 1, corresponding to the patient with a normal neutrophil immunophenotype mentioned above); score of 1, possible neutrophil dysplasia (*n* = 0); score of between 2 and 3, mild neutrophil dysplasia (*n* = 3; 21%); score between 4 and 6, moderate neutrophil dysplasia (*n* = 9; 64%); and score over 6, severe neutrophil dysplasia (*n* = 1; 7%). Thus, assuming a cut-off of > 1 points we correctly classified 13 out of 14 LR-MDS patients and 14 out of 14 controls, given a sensitivity of 93% and a specificity of 100% for the diagnosis of MDS.

#### Monocyte score type 1

The monocytic score type 1 obtained in LR-MDS patients ranged from 0 to 2 (median 1; mean ± SD of 1 ± 1), whereas the score observed in controls ranged from 0 to 1 (Table 6). The total score rank for these groups was of 15 and 2, respectively. Using this score, four groups of MDS patients were identified: score of “0”, no monocyte dysplasia (*n* = 2; 14%); score of 1, possible monocyte dysplasia (*n* = 8; 57%); and score of 2, monocyte dysplasia (*n* = 4; 29%). Comparatively, 12 out of 14 controls (86%) had a score of “0”, and the remaining 2 cases had a score of 1, due to moderate decrease in the fraction of CD14^+^CD16^+^ monocytes (4.9% and 4.1% of total monocytes, respectively). Consequently, assuming a cut-off of ≥1 points we correctly categorized 12 out of 14 LR-MDS patients and 12 out of 14 controls, given a sensitivity and a specificity of 86% for the diagnosis of MDS.

#### Myeloid score type 1

Considering the total myeloid score type 1, obtained by the sum of the correspondent neutrophil and monocytic scores, we found a score of 5 ± 2 for LR-MDS (median of 6, ranging from 0 to 8), while all controls had a score of 1 or under (Table 6). According to this score, four groups of MDS patients were identified: score of “0” (no myelodysplasia) (*n* = 1; 7%, corresponding to the patient mentioned above); score of 1 (possible myelodysplasia) (*n* = 0); score of between 2 and 4 (*n* = 3; 21%) (mild myelodysplasia); score between 5 and 7 (moderate myelodysplasia) (*n* = 7; 50%); and score over 8 (severe myelodysplasia) (n = 3; 21%). In contrast, in the control group, most of the individuals had a score of “0”, with only 3 (21%) having a score of 1. Therefore, assuming a cut-off of ≥2 points we were able to correctly classify 13 out of 14 LR-MDS patients and 14 out of 14 controls, given a sensitivity of 93% and a specificity of 100% for the diagnosis of MDS.

### Immunophenotypic scoring schema 2

#### Neutrophil score type 2

The mean neutrophil score type 2 obtained in LR-MDS patients was of 2 ± 1, ranging from 0 to 3 (total score rank = 28), while all controls had a score of “0” (Table 7). According to this score, four groups of MDS patients were identified: score of “0”, no dysplasia (n = 1; 7%); ii) score of 1 (*n* = 2; 14%); score between 2 and 3 (n = 1; 7%); score between 4 and 6 (n = 0); and score over 6 (n = 0). Thus, similarly to that observed with the neutrophil scoring system type 1, assuming a cut-off of > 1 points we were able to correctly classify 13 out of 14 LR-MDS patients and 14 out of 14 controls (sensitivity of 93% and specificity of 100% for the diagnosis of MDS).

#### Monocyte score type 2

The mean monocyte score type 2 obtained in LR-MDS patients was of 1 ± 1, ranging from 0 to 2 (total score rank = 12), whereas the score observed in controls was of “0” in all cases (Table 7). According this score, four groups of MDS patients were identified: score of “0” (*n* = 5; 36%); score of 1 (*n* = 6; 43%); and score of 2 (n = 3; 21%). Comparatively, all controls had a score of “0”. Consequently, assuming a cut-off of ≥1 point we were able to correctly classify 9 out of 14 LR-MDS patients and all controls, given a sensitivity and a specificity of 64% for the diagnosis of MDS.

#### Myeloid score type 2

Considering the total myeloid score type 2, i.e. the sum of the neutrophil and monocytic scores type 2, we obtained a score of 3 ± 1 for LR-MDS (median of 3, ranging from 0 to 8, total score rank = 39), while all controls had a score of 1 or under (Table 7).

As a result, the scoring schemas type 2 apparently do not offer advantages for the diagnosis of myelodysplasia, as compared to the scoring schemas type 1, which are more easily to calculate.

### MDS thermometers

**Fig. 6:**
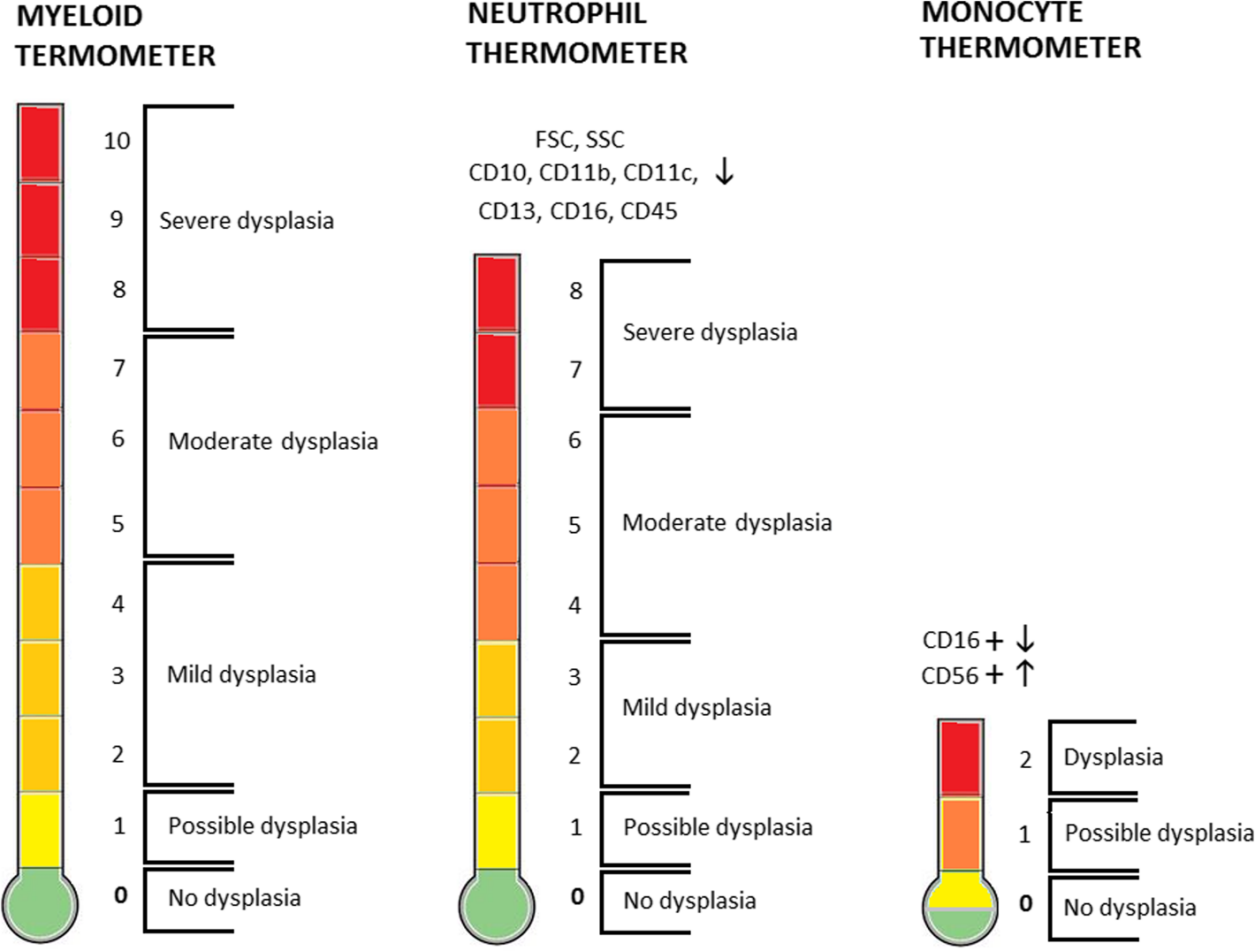
MDS Thermometers. The neutrophil thermometer is based on the neutrophil score system type 1, i.e., the number of cell surface markers abnormally low expressed in PB neutrophils, among 8 parameters considered (FSC, SSC, CD10, CD11b, CD11c, CD13, CD16 and CD45). The monocyte thermometer is based on the monocyte scoring system type 1, i.e., increased fraction of CD56^+^ cells (aberrant CD56 expression) and decreased fraction of CD16^+^ cells (depletion of proinflammatory monocytes) among total PB monocytes. The myeloid thermometer was obtained by the sum of the neutrophil and the monocyte type 1 scores. Abbreviations: FSC, forward scatter; PB, peripheral blood; SSC, side scatter

The immunophenotypic scoring schemas type 1 were used to conceive the MDS Thermometer tool, aimed for the screening of MDS in PB samples in clinical practice, which can be deployed on two thermometers, for neutrophils and monocytes, respectively (Fig. 6). The neutrophil thermometer is based on the neutrophil score system type 1, i.e., the number of cell surface markers abnormally low expressed in neutrophils, among 8 parameters considered (FSC, SSC, CD10, CD11b, CD11c, CD13, CD16 and CD45). The monocyte thermometer is based on the monocyte scoring system type 1, i.e., increased fraction of CD56^+^ cells (aberrant CD56 expression) and decreased fraction of CD16^+^ cells (depletion of proinflammatory monocytes) among total monocytes. The myeloid thermometer was obtained by the sum of the neutrophil and the monocyte type 1 scores.

## Discussion

Progresses made in the last years concerning the assessment of MDS by FCM have led to consensus recommendations for the integration of FCM data in the diagnostic work-up of MDS [21]. However, establishing the diagnosis still requires BM aspirate and biopsy, which are invasive procedures and not always conclusive, especially in LR-MDS.

Given the frequency of MDS patients with mild cytopenias that would not demand therapeutic intervention, due the fact that most MDS patients are not eligible for intensive treatment schedules, and taking into account the easy access to PB samples, it would be desirable to have a PB assay to help guide the need for invasive BM evaluation. Nonetheless, immunophenotypic studies in the PB from patients with MDS are surprisingly scarce. Using the PubMed and applying the key words “flow cytometry”, “myelodysplastic syndromes”, and “peripheral blood”, we found only five studies performed in the PB [27–31].

Cherian et al. (2005) observed that neutrophils from MDS patients had lower SSC and higher expression of CD66 and CD11a than did controls; in some cases, PB neutrophils also displayed abnormal CD116 and CD10 expression [27, 28]. Using these markers, they proposed a score that allowed to distinguish MDS patients from normal controls with a sensitivity of 73% and a specificity of 90% [28].

Some years later (2012), using 3-color FCM, Rashidi et al. observed that CD10 expression on PB neutrophils was significantly decreased in patients with HR-MDS and CMML compared to both non-MDS patients with pancytopenia and to LR-MDS patients [30]. In contrast, they found no significant differences in CD11b, CD13, CD14, CD16, CD33, CD56, and CD64 expression in neutrophils and monocytes from the mentioned groups of patients.

More recently (2013), Meyerson et al. realized that low CD177 expression was frequent in PB and BM neutrophils from patients with clonal myeloid disorders; these findings were most pronounced in MDS, with 52% of cases containing less than 40% of CD177^+^ neutrophils [31].

Altered immunophenotypic features of PB platelets, consisting of abnormal light scatter characteristics, over or under expression of platelet glycoproteins and asynchronous CD34 expression were also described in patients with MDS [29].

Our pilot study revealed, for the first time, that PB neutrophils from LR-MDS not only frequently have decreased FSC and/or SSC, but also commonly display abnormally low levels of CD11b, CD11c, CD10, CD16, CD13 and CD45 expression, as compared to normal neutrophils. The fact that 93% of these patients had abnormally low levels of 2 or more (out of 9) cell surface molecules on neutrophils, as compared to only 29% having abnormal morphology, clearly indicates that FCM is more sensitive for the detection of myelodysplasia in the PB than cytomorphology. In addition, we observed a marked deficiency in proinflammatory CD16^+^ monocytes and a high frequency of CD56 expression in circulating monocytes from patients with LR-MDS, irrespectively of the monocyte counts.

Human PB monocytes have been divided into distinct subsets, referred to as classical (CD14^+^CD16^−^) and non-classical (CD14^+^CD16^+^) monocytes; in between, there are “intermediate” monocytes, which are transitional cells – for review see [45–48]. CD14^+^CD16^+^ monocytes, which were first described in the late 80 [49], account for about 10% of total PB monocytes in healthy adults and appear to be more mature; they express lower levels of CD14 and higher levels of HLA-DR (CD14^+low^CD16^+^HLA-DR^+high^) as compared with classical (CD14^+high^CD16^−^HLA-DR^low^) monocytes [50], and they have distinct patterns of cytokines and chemokine receptors. Specifically, CD14^+^CD16^+^ monocytes have been shown to efficiently produce tumor necrosis factor (TNF), while they produce no or less of the anti-inflammatory cytokine interleukin (IL)-10 [51, 52]. In opposition to classical monocytes, they lack surface expression of CC chemokine receptor type 2 (CCR2), the receptor for monocyte chemotactic protein-1 (MCP-1), and have higher surface expression of CCR5, the receptor for macrophage inflammatory protein 1 alpha (MIP-1alpha) /regulated on activation, normal T cell expressed and secreted (RANTES) chemokine [53]. Their numbers are increased in various pathological conditions, such as HIV infection [54], sepsis [55], inflammatory bowel diseases [56], other inflammatory and autoimmune conditions [57], and tumors [58, 59], and they have been associated with acute or chronic inflammation. In addition, the relative and absolute numbers of the ‘non-classical’ CD14^+^CD16^+^ monocytes increase with the age [60].

Herein we described, for the first time, a depletion of proinflammatory (CD14^+^CD16^+^) monocytes, with consequent increase in the fraction of classical (CD14^+^CD16^−^) monocytes, in the PB of LR-MDS patients. This finding may explain the abnormally low levels of CD11c, CD16 and HLA-DR expression we observed in PB monocytes from patients with LR-MDS. The reasons for this abnormal repartition of the PB monocyte subsets observed in patients with MDS are not clear. Patients included were not medicated with corticosteroids, which are known to induce a selective depletion of the CD14^+^CD16^+^ monocytes [61]. Also, it cannot be explained by aging, as in healthy individuals, proinflammatory CD14^+^CD16^+^ monocytes significantly increase with age, as we observed in our control group. Maybe this is a consequence of a defective monocyte maturation, as already described for CMML [62]. Curiously, Selimoglu-Buet et al. have recently found an increase in the fraction of classical CD14^+^CD16^−^ monocytes, in the PB from patients with CMML, as compared to healthy individuals and to patients with reactive monocytosis [18]. Taking in account that, as stated above, an opposite abnormal repartition of the mentioned monocyte subsets, with increased fractions of CD16^+^ monocytes, has been described in inflammatory and autoimmune conditions [56, 57], infections [54, 55, 57, 63], and cancer [58, 59], the selective depletion of CD14^+^CD16^+^ monocytes in the PB would probably be important for the differential diagnosis between MDS and non-MDS cytopenias.

Abnormal CD56 expression in BM monocytes has already been described in patients with MDS, being observed with higher frequency in HR-MDS, as compared to LR-MDS [64], and is also a frequent in CMML [17, 20, 64], although these findings are not completely understood.

In healthy individuals, around 10% of the monocytes co-express CD56, with the majority of CD56^+^ monocytes being CD14^+high^ [65, 66]. CD56^+^ monocytes are expanded in aging individuals as well as in patients with autoimmune and inflammatory conditions, such as rheumatoid arthritis and inflammatory bowel diseases [66, 67]. Compared to CD56^−^ monocytes, CD56^+^ monocytes spontaneously produce more reactive oxygen intermediates and, upon stimulation, they are stronger producers of cytokines, such as TNF, IL-10 and IL-23 [66]. As so, CD56 expression was recognized as a signal of monocyte activation and/or immunosenescence [66]. Considering the simultaneous decreased fractions of CD14^+low^CD16^+^ and increased fractions of CD14^+high^CD56^+^ monocytes observed in MDS patients, we postulate that blockage of differentiation of classical (CD14^+high^CD16^−^) into proinflammatory (CD14^+low^CD16^+^) monocytes leads to accumulation of the first monocyte population in the PB, which becomes senescent and then acquire CD56 expression.

The phenotypic aberrancies observed in the PB from patients with LR-MDS are consistent with those that have been previously described in the BM, and the 2-tubes/8-color panel we proposed for the screening of myelodysplasia in the PB would allow to evaluate most of the aberrant immunophenotypic features that have been suggested being included in BM studies for the diagnostic work-up of patients with MDS [13, 21]. The exceptions are lineage infidelity markers and some myeloid-associated markers, which are not evaluated with our protocol. In addition, our FCM panel can also be used to quantify and characterize the immature CD34^+^ cells, both in the PB and BM samples. For the evaluation of the myeloid and lymphoid compartments in CD34^+^ BM cells, CD19 may be used instead of CD15, as this marker did not prove to be useful for the diagnosis of neutrophil dysplasia.

Several FCM scoring schemas have been already proposed for diagnosis and prognosis evaluation in MDS, most of them based on the immunophenotypic features of the BM blast cells and on the abnormal immunophenotypic patterns found in maturing myeloid cells [10, 12, 68]. However, due to the complexity of BM analysis, these schemas are difficult to apply in routine clinical practice. To become clinically applicable, FCM should be not only sensitive and specific, but also reproducible, and the results should be easily understood by clinicians. Therefore, our study fills a gap and refine the accuracy to detect myelodysplasia in the PB.

The visual analogue scale we propose for the screening of myelodysplasia in PB samples – the MDS thermometer, is simple, intuitive and easy to apply. It is based on FCM analysis of 10 parameters, 8 in neutrophils (FSC, SSC, CD10, CD11b, CD11c, CD16, CD45) and 2 in monocytes (CD16, CD56), and it allows to distinguish LR-MDS peripheral blood samples from normal PB samples with a sensitivity of 100% and a specificity of 93%. It could be argued that it does not take into account the severity of the deficiency of each molecule observed in myelodysplastic cells. However, the alternative scoring schema that evaluates this aspect but is more difficult to apply, did not improve the performance of the test; maybe it can be useful in specific cases. With the necessary adaptations, the concept of the MDS Thermometer could probably also be applied to the MDS/MPN, such as CMML, in which monocytic aberrancies are expected to be more pronounced.

There are some limitations to the current study. First, the number of cases studied is small and this study should be considered a pilot study. Secondly, due to the difficulties in finding blood donors older than 60 years, we were not able to pair healthy controls and MDS patients for age, and it could be argued that differences between groups may be age-related; however, the fact that no differences were found in the analyzed parameters when younger and older healthy volunteers were compared, except for a higher fraction of CD16^+^ monocytes in the last group, strongly argue against this possibility. Thirdly, we did not study non-MDS patients with cytopenias. Finally, attention should be given to the cytometer setup, calibration and stability, and each center should establish its own normal reference values, on the basis of the mAbs and experimental conditions used.

## Conclusions

Our pilot study reveals an altered neutrophil immunophenotype, often accompanied by an abnormal monocyte immunophenotype, in the PB from nearly all LR-MDS cases, and suggests that assessment of abnormal antigen expression in PB mature myeloid cells may help to identify patients with LR-MDS. Once translated into a straightforward FCM-based score and converted into a visual analogue scale – MDS thermometer –, these findings can be easily applied in clinical practice. However, due to the low number of cases analyzed, further studies with larger series of patients are needed to confirm our preliminary observations. Furthermore, it would be interesting to evaluate HR-MDS patients, as well as CMML and other MDS/MPN. Additional studies are also required in order to evaluate the specificity of such alterations for the diagnosis MDS, by testing other pathological conditions associated with cytopenias.

## Additional files

Additional file 1: **Table S1.** Clinical and laboratorial characteristics of the study population, at diagnosis. Peripheral blood and BM findings at diagnosis, and previous treatments. (DOCX 18 kb)

Additional file 2: **Table S2.** FSC and SSC values and MedFI of expression of the molecules under study on neutrophils from patients with LR-MDS, as compared to controls. Median (and range) values of the MedFI obtained for each parameter analyzed by FCM in PB neutrophils from LR-MDS patients and healthy controls. (DOCX 14 kb)

Additional file 3: **Table S3.** FSC and SSC values and MedFI of expression of the molecules under study on monocytes from patients with LR-MDS, as compared to controls. Median (and range) values of the MedFI obtained for each parameter analyzed by FCM in PB monocytes from LR-MDS patients and healthy controls. (DOCX 14 kb)

## Abbreviations

APC: Allophycocyanin
BC: Beckman Coulter
BD: Becton Dickinson
BDB: Becton Dickinson Bioscience
BDH: Becton Dickinson Horizon
BL: Biolegend
BM: Bone marrow
CCR2: C-C chemokine receptor type 2
CCR5: C-C chemokine receptor type 5
CMML: Chronic myelomonocytic leukemia
Cy5.5: Cyanine 5.5
Cy7: Cyanine 7
EPO: Erythropoietin
FAB: French-American-British
FCM: Flow cytometry
FITC: Fluorescein isothiocyanate
FSC: Forward scatter
G-CSF: Granulocyte-colony stimulating factor
GM-CSF: Granulocyte monocyte-colony stimulating factor
Hg: Hemoglobin
HR-MDS: Higher risk myelodysplastic syndrome (IPSS: intermediate-2 + high)
IL: Interleukin
IOT: Immunotech
IPSS: International Prognostic Scoring System
IPSS-R: Revised International Prognostic Scoring System
KO: Khrome Orange
LDH: Lactate dehydrogenase
LR-MDS: Lower risk myelodysplastic syndrome (IPSS: low + intermediate-1)
mAb: Monoclonal antibody
MCP-1: Monocyte chemotactic protein-1
MCV: Mean corpuscular volume
MDS: Myelodysplastic syndromes
MDS/MPN: Myelodysplastic/myeloproliferative neoplasms
MDS-del(5q): Myelodysplastic syndromes with isolated deletion of chromosome 5q
MDS-EB: Myelodysplastic syndromes with excess of blasts
MDS-MLD: Myelodysplastic syndromes with multilineage dysplasia
MDS-RS: Myelodysplastic syndromes with ring sideroblasts
MDS-RS-MLD: Myelodysplastic syndromes with ring sideroblasts with multilineage dysplasia
MDS-RS-SLD: Myelodysplastic syndromes with ring sideroblasts with single lineage dysplasia
MDS-SLD: Myelodysplastic syndromes with single lineage dysplasia
MDS-U: Myelodysplastic syndromes, unclassifiable
MedFI: Median fluorescence intensity
MIP-1alpha: Macrophage inflammatory protein 1alpha chemokine
NA: Not applicable
PB: Peripheral blood
PBS: Phosphate buffered saline
PE: Phycoerythrin
PerCP: Peridinin chlorophyll protein
PerCP-Cy5.5: Peridinin chlorophyll protein Cy5.5
PMT: Photomultiplier tubes
RANTES: Regulated on activation, normal T cell expressed and secreted chemokine
RBC: Red blood cells
SSC: Side-scatter
TNF: Tumor necrosis factor
V450: Violet 450
WBC: White blood cells
WHO: World Health Organization
