# Supplementary material for: A pilot study on the usefulness of peripheral blood flow cytometry for the diagnosis of lower risk myelodysplastic syndromes: the “MDS thermometer”
Source: BMC Hematol. 2018 Mar 13;18:6. doi: 10.1186/s12878-018-0101-8 (PMC5850915; doi:10.1186/s12878-018-0101-8)
Supplement: Supplementary file 1 — Table S1. Clinical and laboratorial characteristics of the study population, at diagnosis. Peripheral blood and BM findings at diagnosis, and previous treatments. (DOCX 18 kb) [file 12878_2018_101_MOESM1_ESM.docx]

# A PILOT STUDY ON THE USEFULNESS OF PERIPHERAL BLOOD FLOW CYTOMETRY FOR THE DIAGNOSIS OF LOWER RISK MYELODYSPLASTIC SYNDROMES: THE “MDS THERMOMETER”

# Additional file 1

## Table S1. Clinical and laboratorial characteristics of the study population at diagnosis

| **Peripheral blood cell counts*** |  |
| --- | --- |
| Hemoglobin (g/dL) | 9.1 (7.2-11.9) |
| MCV (fL) | 103 (85-120) |
| MCV > 100 fL | 9/14 (64%) |
| WBC (x10^6^/L) | 4120 (1420-8960) |
| Neutrophils (x10^6^/L) | 2567 (575-7025) |
| Monocytes (x10^6^/L) | 305 (0-1820) |
| Platelets (x10^9^/L) | 200 (61-591) |
| Blasts (%) | 0.0 (0.0-3.0) |
| **Cytopenias and abnormal peripheral blood cell counts**** |  |
| Anemia (<12.5 g/dL) | 14/14 (100%) |
| Neutropenia (< 2000 x 10^6^/L) | 5/14 (36%) |
| Thrombocytopenia (<150 x 10^9^/L) | 4/14 (29%) |
| Monocytosis (>1000 x 10^6^/L) | 2/14 (14%) |
| Thrombocytosis (>500 x 10^9^/L) | 1/14 (7%) |
| Isolated cytopenia | 8/14 (57%) |
| Bicytopenia | 3/14 (21%) |
| Pancytopenia | 3/14 (21%) |
| **Peripheral blood cell morphology** |  |
| Red blood cell abnormalities | 14/14 (100%) |
| Macrocytosis (macrocytes / macro-ovalocytes) | 9/14 (57%) |
| Anisocytosis | 10/14 (71%) |
| Poikilocytosis | 3/14 (21%) |
| Polychromasia | 1/14 (7%) |
| Other RBC abnormalities | 0/14 (0%) |
| Neutrophil abnormalities | 4/14 (29%) |
| Abnormal nuclear segmentation | 4/14 (29%) |
| Hyposegmented nuclei | 4/14 (29%) |
| Pseudo-Pelger-Huet abnormality (bilobed) | 0/14 (0%) |
| Hypersegmented nuclei | 0/14 (0%) |
| Abnormal granulation | 1/14 (7%) |
| Hypogranulated | 1/14 (7%) |
| Abnormal granule distribution (Dohle bodies) | 0/14 (0%) |
| Hypergranulated | 0/14 (0%)- |
| Platelet abnormalities | 4/14 (29%) |
| Platelet anisocytosis | 4/14 (29%) |
| Giant platelets | 2/14 (14%) |
| Hypogranulated platelets | 0/14 (0%) |
| **Biochemistry parameters** |  |
| Serum ferritin levels (ng/mL) | 1731 (35-4193) |
| Increased serum ferritin levels (>454 ng/mL) | 13/14 (93%) |
| Serum LDH levels (IU/L) | 174 (101-901) |
| Increased serum LDH levels (> 225 IU/L) | 3/14 (23%) |
| **Bone marrow findings** |  |
| Cellularity |  |
| Hypocellular | 5/14 (36%) |
| Normocellular | 4/14 (29%) |
| Hypercellular | 5/14 (36%) |
| Myeloid / Erythroid ratio | 1.4 (0.6-3.9) |
| Blasts (% total nucleated cells) | 1.4 (0.3-5.3) |
| Erythroid dysplasia | 13/14 (93%) |
| Granulocytic dysplasia | 4/14 (29%) |
| Megakaryocytic dysplasia | 7/14 (70%) |
| Extracellular iron deposits |  |
| Decreased | 2/14 (14%) |
| Normal | 3/14 (21%) |
| Increased | 9/14 (64%) |
| Ringed sideroblasts |  |
| Presence of RS | 8/14 (57%) |
| Percentage of RS | 30 (0-67) |
| Percentage of RS >15% | 7/14 (50%) |
| Percentage of RS in patients with >15% RS | 54 (30-67) |
| **Cytogenetics** |  |
| Conventional cytogenetics | 6/14 (46%) |
| Normal karyotype | 3/6 (50%) |
| Isolated monosomy 18 | 1/6 (17%) |
| Non clonal aneuploidies | 2/6 (34%) |
| Fluorescence *in situ* hybridization (FISH) | 9/14 (57%) |
| Chromosome 5: del(5q) | 1/9 (11%) |
| Other chromosome abnormalities | 0/8 (0%) |
| Not available | 4/14 (29%) |
| **Treatments***** |  |
| Supportive treatment (e.g. transfusions, growth factors) | 14/14 (100%) |
| Number of patients transfused with RBC | 13/14 (93%) |
| Number of occasionally transfused patients | 2/14 (14%) |
| Number of regularly transfused patients | 11/14 (79%) |
| Number of RBC units / regularly transfused patient | 57 (15-462) |
| Number of RBC units / regularly transfused patient / month | 1.4 (0.3-4.0) |
| EPO therapy before or during the study | 13/14 (93%) |
| EPO therapy during the study | 2/14 (14%) |
| Favorable response to EPO | 3/13 (23%) |
| G-CSF therapy before the study | 5/14 (36%) |
| G-CSF therapy during the study | 0/14 (0%) |
| Favorable response to G-CSF | 0/5 (0%) |

Abbreviations: EPO, erythropoietin; G-CSF, Granulocyte-colony stimulating factor; GM-CSF, Granulocyte monocyte-colony stimulating factor; LDH, lactate dehydrogenase; MCV, mean corpuscular volume; NA, not applicable; RBC, red blood cells; RBC, red blood cells; RS, ringed sideroblasts; WBC, white blood cells.

Results are expressed as absolute and (relative) frequencies, or as median (range) values.

Percentages were approximated to the closest full unit, except for the percentages of blasts, which were rounded to one decimal place.

All the laboratory parameters mentioned in the table were obtained at diagnosis, except for the biochemistry results which were obtained at the time of the study.

* Values at the time of the study: hemoglobin: 8.1 (6.0-11.3); MCV: 95.0 (81.1-111.3); MCV > 100: 6/14 (46%); WBC: 4485 (870-8470); neutrophils: 2590 (365-6945); monocytes: 426 (17-1132); platelets: 175 (37-303); % blasts. 0.0 (0.0-1.0).

** Values at the time of the study: anemia: 14/14 (100%); neutropenia: 6/14 (46%); thrombocytopenia: 6/14 (46%); monocytosis: 1/14 (7%); thrombocytosis: 0/14 (0%); isolated cytopenia: 7/14 (50%); bicytopenia: 2/14 (14%); pancytopenia: 5/14 (36%).

*** None of the patients had received treatment with cytoreductive, hypomethylating and/or immunosuppressive drugs, neither iron chelation therapy or GM-CSF and thrombopoietin receptor agonists.
