# Supplementary material for: A pilot study on the usefulness of peripheral blood flow cytometry for the diagnosis of lower risk myelodysplastic syndromes: the “MDS thermometer”
Source: BMC Hematol. 2018 Mar 13;18:6. doi: 10.1186/s12878-018-0101-8 (PMC5850915; doi:10.1186/s12878-018-0101-8)
Supplement: Supplementary file 2 — Table S2. FSC and SSC values and MedFI of expression of the molecules under study on neutrophils from patients with LR-MDS, as compared to controls. Median (and range) values of the MedFI obtained for each parameter analyzed by FCM in PB neutrophils from LR-MDS patients and healthy controls. (DOCX 14 kb) [file 12878_2018_101_MOESM2_ESM.docx]

# A PILOT STUDY ON THE USEFULNESS OF PERIPHERAL BLOOD FLOW CYTOMETRY FOR THE DIAGNOSIS OF LOWER RISK MYELODYSPLASTIC SYNDROMES: THE “MDS THERMOMETER”

# Additional file 2

## Table S2. FSC and SSC values and MedFI of expression of the molecules under study on PB neutrophils from patients with LR-MDS, as compared to controls.

| **Parameter*** | **Controls**  **(n=14)** | **LR-MDS**  **(n=14)** | **P value**** |
| --- | --- | --- | --- |
| **FSC** | 115 352 (107 079 -123 524) | 97 432 (77 104-130 882) | **0.008** (🠛) |
| **SSC** | 115.148 (87.268 – 141.315) | 79.310 (55 713 – 112 365) | **<0.001** (🠛) |
| **CD10** | 10 432 (6 627 – 15 221) | 5 290 (9 214 – 21 796) | **<0.001** (🠛) |
| **CD11b** | 13 317 (10 303 – 18 374) | 6 424 (3 009 – 20 902) | **<0.001** (🠛) |
| **CD11c** | 4 168 (3 203 – 5 229) | 1 950 (1 462 – 3 977) | **<0.001** (🠛) |
| **CD13** | 6 065 (4 017 – 9 969) | 4.320 (1.309 – 10.475) | **0.022** (🠛) |
| **CD14** | 652 (456 – 1 622) | 654 (217 – 1 507) | 0.435 |
| **CD15** | 5 101 (1 598 – 9 473) | 4 712 (1 778 – 7 533) | 0.435 |
| **CD16** | 31 737 (15 585 – 42 215) | 14 637 (2 200 – 38 439) | **0.002** (🠛) |
| **[CD56]** | 56 (4 – 126) | 56 (18 – 571) | 0.854 |
| **CD64** | 275 (83 – 476) | 211 (60 – 669) | 0.251 |
| **[HLA-DR]** | 71 (18 – 129) | 111 (51 – 209) | 0.012 |
| **CD45** | 2 396 (1.222 – 3.294) | 2.033 (886 – 6427) | 0.081 |

Abbreviations: LR-MDS, lower risk myelodysplastic syndromes; FSC, Forward scatter; MedFI, median fluorescence intensity (arbitrary units); SSC, side scatter; PB, peripheral blood.

Results are expressed as median (range) values of the MedFI obtained for each parameter.

Values were approximated to the closest full unit.

* Markers found to be absent on neutrophils are shown in square brackets.

** Mann-Whitney U test. Arrows inside parenthesis: (🠛) decreased expression.
