# Supplementary material for: A pilot study on the usefulness of peripheral blood flow cytometry for the diagnosis of lower risk myelodysplastic syndromes: the “MDS thermometer”
Source: BMC Hematol. 2018 Mar 13;18:6. doi: 10.1186/s12878-018-0101-8 (PMC5850915; doi:10.1186/s12878-018-0101-8)
Supplement: Supplementary file 3 — Table S3. FSC and SSC values and MedFI of expression of the molecules under study on monocytes from patients with LR-MDS, as compared to controls. Median (and range) values of the MedFI obtained for each parameter analyzed by FCM in PB monocytes from LR-MDS patients and healthy controls. (DOCX 14 kb) [file 12878_2018_101_MOESM3_ESM.docx]

# A PILOT STUDY ON THE USEFULNESS OF PERIPHERAL BLOOD FLOW CYTOMETRY FOR THE DIAGNOSIS OF LOWER RISK MYELODYSPLASTIC SYNDROMES: THE “MDS THERMOMETER”

# Additional file 3

## Table S3. FSC and SSC values and MedFI of expression of the molecules under study on PB monocytes from patients with LR-MDS, as compared to controls.

| **Parameter*** | **Controls**  **(n=14)** | **LR-MDS**  **(n=14)** | **P value*** |
| --- | --- | --- | --- |
| **FSC** | 97 821 (89 536 – 114 799) | 95 396 (84 796 – 112 951) | 0.358 |
| **SSC** | 43 966 (39 162 – 51 885) | 39 271 (36 032 – 51 460) | 0.098 |
| **CD10** | 350 (185 – 1 385) | 596 (115 – 1 237) | 0.188 |
| **CD11b** | 8 243 (4 892 – 12 286) | 6 146 (1 785 – 14 254) | 0.089 |
| **CD11c** | 8 334 (5 205 – 13 876 | 5 955 (3 037 – 10 132) | **0.004 (**🠛) |
| **CD13** | 7 005 (981 – 14 084) | 7 020 (515 – 14 502) | 0.818 |
| **CD14** | 16 722 (10 256 – 25 823) | 17 791 (9 535 – 27 655) | 0.435 |
| **CD15** | 243 (123 – 502) | 297 (99 – 488) | 0.335 |
| **CD16** | 229 (132 – 622) | 163 (105 – 245) | **0.005 (**🠛) |
| **CD56** | 252 (156 – 409) | 376 (189 – 6 861) | **0.006 (🠙)** |
| **CD64** | 5 786 (3 843 – 8 941) | 6 522 (2 384 – 14 165) | 0.646 |
| **HLA-DR** | 7 910 (5 619 – 11 889) | 6 532 (1 588 – 11 363) | **0.042 (**🠛) |
| **CD45** | 4 147 (3 172 – 6 230) | 4 049 (2 136 – 5 615) | 0.215 |

Abbreviations: LR-MDS, lower risk myelodysplastic syndromes; FSC, Forward scatter; MedFI, median fluorescence intensity (arbitrary units); SSC, side scatter; PB, peripheral blood.

Results are expressed as median (range) values of the MedFI obtained for each parameter.

Values were approximated to the closest full unit.

* Mann-Whitney U test. Arrows inside parenthesis: (🠛) decreased expression; (🠙) increased expression.
